# Health inequalities in cancer care: a literature review of pathways to diagnosis in the United Kingdom

**DOI:** 10.1016/j.eclinm.2024.102864

**Published:** 2024-09-27

**Authors:** Emily C.S. Scott, Peter J. Hoskin

**Affiliations:** a Mount Vernon Cancer Centre, London, United Kingdom; b Division of Cancer Sciences, University of Manchester, Manchester, United Kingdom; c East of England Cancer Alliance, Ely, United Kingdom

**Keywords:** Inequality, Cancer, Diagnosis, Barriers, Health literacy

## Abstract

This literature review discusses current health disparities in cancer care in the United Kingdom, spanning access to services, diagnosis, and outcomes. These inequities stem from a complex interplay of factors such as health literacy, ethnicity, socioeconomic status, age, gender, geography, and lifestyle choices. Health literacy plays a crucial role in timely healthcare seeking and diagnosis, while cultural beliefs significantly shape perceptions and behaviours. Socioeconomic barriers often result in delayed diagnosis and inferior outcomes due to limited access to preventive measures and high-quality treatment. Barriers to timely diagnosis include non-specific symptoms, variations in diagnostic intervals influenced by age and gender, and non-attendance at secondary care appointments. Addressing these challenges necessitates initiatives aimed at improving health literacy, implementing culturally sensitive screening approaches, and enhancing accessibility to both primary and secondary care services.

## Introduction

Despite record-high cancer survival rates in the United Kingdom (UK), its performance lags behind other developed nations with similar healthcare systems, indicating existing disparities in cancer survival outcomes.^1^, ^2^, ^3^, ^4^ These discrepancies are, in part, a result of unequal access to and utilisation of cancer services, reflecting broader health inequalities in the United Kingdom.^5^ Cancer health inequalities encompass disparities in cancer incidence, outcomes, and healthcare access among different demographic groups, influenced by factors such as health literacy, ethnicity, socioeconomic status (SES), age, gender, geography, screening practices, healthcare access, and lifestyle choices.^6^ However, health inequalities often intersect, a concept known as intersectionality, where overlapping social identities—such as age, ethnicity, gender, and socioeconomic status—combine to create unique and compounded disadvantages in accessing and receiving quality healthcare.^6^ For example, people from all ethnic minority groups, except Indian, Chinese, White Irish and White Other groups, are more likely than White British people to live in the most deprived decile in England.^7^

Disparities in health literacy can lead to delayed presentation to healthcare services, resulting in late-stage diagnoses and limited treatment options. Lower health literacy is more commonly found in those from ethnic minorities and from more deprived areas.^8^, ^9^, ^10^, ^11^, ^12^

Ethnic minority groups experience variations in cancer incidence and outcomes due to genetics, cultural differences, and SES within their communities.^13^^,^^14^ Interpreting cancer statistics is challenging due to multiple differing models used to capture ethnicity data leading to variation in the categorisation and interpretations of ethnicity, and multiple entries of ethnicity for a single individual which do not always align.^14^^,^^15^ We must gain consensus on universal ethnicity definitions, as the percentage of people from ethnic minority populations in the UK is expected to increase from 8% to 20% by 2051.

Socioeconomic challenges often result in higher cancer incidence and poorer outcomes among individuals from more deprived backgrounds, attributed to limited access to preventive measures, delayed diagnosis, and reduced access to high-quality treatment.^6^

Age-related barriers, such as physical frailty, cognitive decline, and mobility challenges, can impede timely access to healthcare services for older adults. These barriers are often compounded by ageist attitudes within the healthcare system, where such perceptions can lead to delayed diagnoses, reduced treatment options, and a lack of proactive care. In addition, logistical issues, such as difficulty in traveling to medical appointments or navigating complex healthcare systems, further exacerbate these challenges.

There are significant inequities in healthcare for the Lesbian, Gay, Bisexual, Transgender, Queer∖Questioning (LGBTQ+) community, which is often overlooked in research. Diagnosis and treatment may be affected by knowledge gaps and attitudes within the healthcare system, and individuals may also delay help-seeking due to past negative experiences, fear of stigma, or concerns about confidentiality.^16^

Regional disparities in cancer rates and outcomes persist across England, with worse outcomes observed in the North compared to the South.^6^ Access to healthcare services, screening programmes, and lifestyle factors vary between urban, rural and coastal areas. Unequal participation in cancer screening programmes leads to disparities in early detection, and variations in access to specialised cancer treatment facilities and services may contribute to disparities in outcomes.

Lifestyle-related factors, such as smoking, diet, alcohol consumption, and level of physical activity, contribute significantly to variations in cancer rates, with socioeconomic challenges, like poverty and debt, exacerbating unhealthy behaviours.^6^^,^^17^ A review from 2017 showed those with three or more behavioural risk factors made up 27% of the population in the most deprived quintile, compared to 14% in the most affluent.^5^ The poorest 10% of UK households need to spend 74% of their income on food to follow the Government's healthy diet guidance, compared to only 6% of income from the richest 10%.^18^

Health inequalities are a critical injustice within the National Health Service (NHS), prompting the introduction of the Core20PLUS5 initiative: a targeted strategy by the NHS in England to reduce health inequalities, particularly focusing on the most deprived 20% of the population, known as the Core20.^19^ Within cancer care, the initiative emphasises improving early cancer diagnosis among these disadvantaged groups, who often face significant barriers to accessing timely screenings and treatments. In addition to the Core20, the framework also addresses other marginalised populations, such as ethnic minorities and people with disabilities, who similarly experience disparities in cancer outcomes. By focusing on these groups, the Core20PLUS5 initiative aims to increase early detection and improve cancer survival rates across the board, ensuring that all individuals, regardless of their background, receive equitable cancer care.

The Andersen Model is the framework used to structure this review. It theorises the stages of delay in seeking treatment and is widely used to study delays in cancer diagnosis.^20^ It identifies stages like appraisal delay (time taken to recognise a symptom as serious), illness delay (time from recognising illness to deciding to seek care), behavioural delay (time between deciding to seek care and acting on it), scheduling delay (time taken to get an appointment), and treatment delay (time from first consultation to treatment initiation). A systematic review applying the Andersen Model to cancer diagnosis revealed significant appraisal and treatment delays. Illness delay was sometimes indistinguishable from appraisal delay, while behavioural delay was less evident. The review highlighted the need for more consistent definitions and methods in research to allow better comparisons across studies. The Anderson Model of diagnostic delay will be used in this review to examine patient pathways to diagnosis of cancer. Cancer screening is discussed at the end of the Anderson Model.

## Search strategy and selection criteria

A literature search was performed in June 2024 using the OVID platform to search Medline and EMBASE databases. Search terms included ‘health inequality’ OR ‘health inequity’ AND ‘cancer’ OR ‘oncology’ AND ‘United Kingdom’ OR ‘UK’. After adjusting for papers published since 2013, 1160 records were found. Reviewing titles, studies conducted outside the UK were excluded due to the differences in insurance and healthcare systems, as were abstracts, editorials, interviews, news, notes, and letters. 936 abstracts were assessed where duplicates, studies outside the UK, and those out of scope were excluded.

**Fig. 1:**
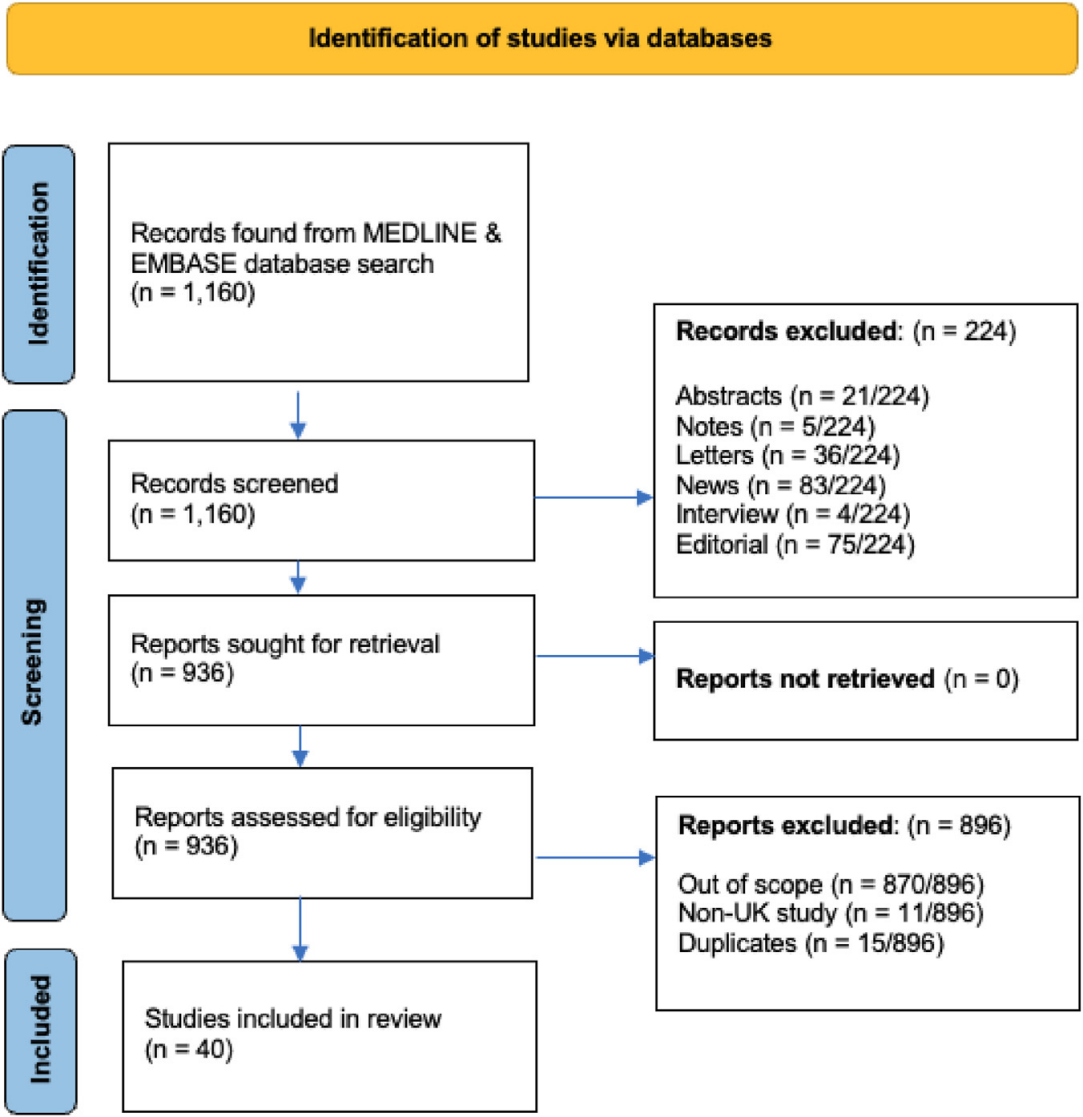
*PRISMA flowchart of study identification and inclusion criteria*.

A total of 40 papers were identified and summarised in this literature review of health inequalities on patient appraisal, help-seeking behaviour and pre-diagnosis of patients with cancer in the UK. A summary of the search strategy and inclusion and exclusion criteria is shown in Fig. 1.

## Appraisal delay

This stage represents the time individuals take to identify a serious symptom, influenced by factors such as personal experience, health literacy, demographics, cultural beliefs, social support networks, and health campaigns. Evidence shows that delays in the cancer care pathway often stem from this stage, highlighting the importance of individuals’ understanding of symptom characteristics and their ability to attribute them to a serious condition.^20^

Research indicates that over one third of the adult population, particularly those in deprived areas or older age groups, lacks the necessary health literacy to effectively manage their own health.^8^ Health literacy, an increasingly acknowledged facet in healthcare and a growing national priority, encompasses the ability of individuals to access, comprehend, evaluate and apply information crucial for making informed healthcare decisions. Within the realm of cancer care, it hinges on one's understanding of cancer symptoms, awareness of available screening options, and familiarity with healthcare systems such as the NHS. This level of comprehension significantly influences the timing of healthcare seeking and the stage at which cancer is diagnosed, crucial elements given the complex decisions individuals face during diagnosis and treatment, often amidst physical and emotional distress.^21^^,^^22^ A review of National Cancer Control Plans (NCCPs) across Europe, including the UK, underscores the pivotal role of health literacy. NCCPs, integral to public health initiatives aimed at reducing cancer incidence and mortality, align closely with the NHS long-term plan and Public Health England's focus on health literacy.^23^, ^24^, ^25^

A large study of cancer awareness and survival showed that those with the lowest awareness of cancer symptoms were in the most socioeconomically deprived areas. Having a higher cancer symptom awareness correlated with better cancer survival indices with each additional symptom recognised associated with a 1.56% increase in the cancer survival index.^9^

A study utilising telephone interviews with over 7000 individuals across six countries, including the UK, focused on the perception of cancer symptoms and their influence on timely presentation to primary care.^3^ The findings showed when symptoms were not perceived as potentially indicative of cancer, respondents delayed help-seeking for over two weeks regardless of demographics or healthcare access. Notably, men, individuals from lower socioeconomic backgrounds, and people from certain ethnic groups demonstrated poorer awareness, consistent with prior research highlighting the correlation between late-stage diagnoses and these demographic factors.^10^^,^^11^ The review, however potentially inflated its results due to shared method variance highlighted in its methodology.

Certain cancers, such as ovarian, pancreatic and blood cancers, present with vague, gradual and nonspecific symptoms, often leading to delayed diagnosis and lower survival rates, particularly when diagnosed via emergency presentations. Blood cancers, including leukaemia, lymphoma and multiple myeloma, are frequently diagnosed at later stages, with low symptom recognition contributing to these delays.^26^^,^^27^ The Blood Cancer Awareness Measure evaluates public awareness and primary care barriers for blood cancer, with disparities in symptom awareness and healthcare-seeking behaviours.^26^ A study assessed participants’ knowledge of blood cancer symptoms, and while 52% of individuals reported potential blood cancer symptoms, half of this cohort did not contact their GP, and only 7% linked the symptoms to cancer, highlighting the need for improved public education and access to primary care services.

To enhance early cancer diagnosis and survival rates, the UK Government prioritises increasing public cancer awareness, particularly among adolescents who face communication barriers with doctors and parents.^28^ A cluster randomised control trial utilised school-based psycho-educational interventions to improve cancer symptom awareness and communication. The intervention group improved symptom recognition and communication about cancer among adolescents, although concerns remain regarding long-term sustainability and impact. However, if this could be included in routine school education, and re-visited at certain points during early education, it could have substantial and long-lasting impact on general health, cancer prevention and early cancer detection.

## Illness and behavioural delay

Illness delay refers to the period between when an individual first recognises that they are unwell and when they decide to seek medical assistance. This delay is influenced by personal beliefs about the effectiveness of healthcare, trust in the healthcare system, past experiences with healthcare, and cultural factors such as fear, stigma and concerns about finances, work and caregiving responsibilities. While illness delay generally contributes less to diagnostic delays compared to appraisal delay, it remains significant as some patients may misattribute symptoms to benign conditions or postpone seeking help after acknowledging their illness.^20^

Following the recognition of illness, behavioural delay describes the time between deciding to seek medical help and actually doing so. This delay can be affected by personality traits, the perceived severity of symptoms, trust in the healthcare system, competing life priorities, transportation issues, appointment availability and cultural influences. Although behavioural delay is generally less impactful on diagnostic timelines compared to appraisal delay, it is still relevant. The decision to seek medical help and the action to follow through are often closely connected, but various factors can still cause additional delays in receiving timely care.^20^

Ethnicity and cultural factors play a significant role in shaping attitudes and behaviours related to cancer. A systematic review examining cancer beliefs among minority populations in the UK revealed insights from qualitative studies, shedding light on emotional reactions, knowledge, belief and interactions with healthcare services.^29^ Delayed diagnoses are compounded by pessimism, fear and cultural nuances like health secrecy and stigma. For instance, disclosing certain health conditions might bring shame or stigma to an individual or their family, so maintaining secrecy is seen as vital to preserving dignity and social standing. Barriers within healthcare services, including language obstacles and reliance on traditional medicine, further exacerbate the challenge. Research has shown that stigma related to cancer is related to a women's country of birth, and how long she has lived in the UK.^30^^,^^31^

A cross-sectional survey explored cancer fear and fatalism among White, Caribbean, African, Indian, Pakistani and Bangladeshi women.^32^ Cancer fear and fatalism were found to be higher among individuals with lower education levels. Indian and African women exhibited the highest fear, while Bangladeshi women reported the lowest. Fatalistic views were more prevalent among all minorities compared to White individuals. Less acculturated women were less likely to worry about getting cancer but more likely to feel unease when considering it. Additionally, previous studies found that women, those with lower education, and ethnic minorities experienced greater cancer fear and fatalism.^23^^,^^33^

A qualitative study on Black men in the UK revealed a lack of awareness regarding heightened prostate cancer risk.^34^ People reported a believed association between cancer and processed foods, or considering it a ‘White people thing,’ while one viewed it as a government population reduction tactic. Cultural resistance to intimate examinations, driven by masculinity beliefs, and varying opinions on body size, with higher body mass index signifying affluence, further complicate health perceptions. The study revealed cultural meanings attributed to behaviours like smoking. Cannabis use by some is seen as meditative and ‘healthy’ with the right mindset, and tobacco smoking signifies higher social status.^34^ Rastafarian belief in eternal life further complicates health perceptions and hinders proactive healthcare seeking. ‘Babylon,’ a term used to signify oppression, is sometimes used negatively to describe Western medicine, contributing to reluctance in seeking cancer treatment. Concerns about side effects, especially impacting quality of life and sexual function, and suspicions about the NHS being a tool for control, further hinder engagement with healthcare. This study highlights a notable gap in engaging Black men with health services, suggesting avenues for improvement through community involvement and collaboration with cultural leaders.

Understanding socioeconomic disparities is essential in addressing disparities in cancer outcomes. Varied attitudes toward cancer within different socioeconomic groups influence screening uptake, with lower SES individuals often exhibiting more fatalistic views.^35^ A study assessing views on cancer in a population sample grouped by education, revealed that those with lower education reported more negative attitudes, with 57% agreeing that ‘treatment is worse than cancer’ and 27% believing ‘cancer is a death sentence'.^36^ Such fatalistic beliefs may result in missed screenings or delayed help-seeking behaviour, as individuals may perceive no benefit in early cancer detection if they believe that a cancer diagnosis inevitably leads to death. The study's reliance on education as the sole SES measure, warrants caution in drawing definitive conclusions about the relationship between SES and attitudes.

Certain types of cancer may initially present with symptoms that do not significantly disrupt daily functioning. For instance, post-menopausal bleeding, while a potential red flag for cancer, may not immediately impact quality of life until advanced stages. Moreover, individuals' interpretations of concerning signs can vary widely; symptoms like a persistent cough or an atypical breast lump might not trigger immediate concern. Psychosocial factors play a significant role in patient-mediated delays in seeking healthcare, encompassing perceptions of symptom seriousness, fear of cancer, embarrassment and concerns about wasting time.^37^, ^38^, ^39^

A population-based survey highlighted that embarrassment regarding symptoms, especially concerning breast or bowel issues, emerged as a prominent barrier, while concerns about wasting time were more pronounced in cases of vague lung or ovarian cancer symptoms.^2^ Univariate analysis indicated that younger age, perceived difficulty in accessing healthcare, or possessing tertiary qualifications were associated with delayed primary care presentation. Stigma and self-blame further compounded delays in seeking medical attention for symptoms indicative of lung cancer, a phenomenon well-documented in literature.^40^^,^^41^ However, the underrepresentation of men, individuals from lower socioeconomic backgrounds, and people from ethnic minorities limits the comprehensiveness of the analysis concerning the role of these disparities in late presentations.

An investigation in adults over 50 revealed that symptom persistence, cancer awareness, and social influence positively influenced health-seeking behaviour.^4^ Negative attitudes, stoicism, symptoms trivialisation, lack of confidence in the healthcare system, and fear act as significant barriers. The study was limited to a London-based population with a small sample, lacking diversity in age, ethnicity, and SES, and so may have restricted generalisability.

## Scheduling delay

This period encompasses the time between when an individual decides to seek medical assistance and when they attend a first appointment. Several factors can influence this delay, including the desire to see a specific specialist, the perceived urgency of the medical issue, personal circumstances, appointment availability, the complexity of the scheduling process, geographical accessibility, and financial considerations. Evidence on the impact of scheduling on diagnostic delays is mixed, with varying conclusions about its significance.^20^

Numerous studies in the UK have underscored the hurdles in accessing General Practice (GP) appointments as a significant deterrent to timely healthcare seeking.^37^, ^38^, ^39^^,^^42^ Scheduling delays are also compounded by an individual's understanding of the NHS system, and ability to navigate telephone or digital triage systems, both of which are essential for accessing primary, or more complex secondary care appointments. This adds another layer of difficulty to timely help-seeking.^21^^,^^22^^,^^43^ With the widespread adoption of triage systems post-pandemic, further research needs to review their impact on health inequalities when accessing primary care appointments, as little has been done to evaluate this since their implementation.

A study reviewed inequalities in patient experience within NHS primary care between 2011 and 2017, focusing on sociodemographic characteristics such as age, sex, ethnicity, sexual orientation, deprivation and geographical region.^44^ Results showed that younger patients, ethnic minorities, those in deprived areas, and sexual minorities consistently reported less positive experiences across various dimensions of care including GP access. While inequalities remained relatively stable over time, there was a notable decline in patient experience of access to care, particularly among those in more deprived areas. Additionally, older patients experienced a significant reduction in continuity of care.

A population-based survey conducted in the UK revealed a good awareness of cancer symptoms, yet it also identified a higher prevalence of perceived healthcare barriers compared to other countries included in the study.^2^ The analysis, encompassing lung, breast, bowel and ovarian cancers, highlighted that perceived barriers significantly contribute to delays in seeking primary care, particularly when patients perceive difficulties in accessing their GP easily. Non-specific symptoms such as those seen in ovarian, pancreatic and blood cancers also pose challenges in accessing primary care, contributing to these delays.^27^

## Treatment delay

Treatment delays encompass the interval between the first medical appointment and the initiation of treatment. There is strong evidence that treatment delay is a critical issue in cancer care. Contributing factors include healthcare provider shortcomings such as inadequate symptom investigation, inappropriate treatment for non-cancerous conditions, lack of follow-up, and referral delays. These delays are frequently exacerbated by patient-related factors and systemic inefficiencies, particularly in cancers like ovarian cancer, which is notoriously difficult to diagnose. Additionally, treatment delay is now understood to involve multiple stages, including obtaining test results, securing specialist appointments, and receiving pathology reports. The process can be iterative, with reappraisal of symptoms potentially causing further delays. Both healthcare provider practices and systemic issues are crucial in determining the extent of treatment delays.^20^

Non-specific symptoms can pose challenges in diagnosing cancer, particularly in teenagers and young adults (TYAs), leading to a higher number of consultations before referral to secondary care.^45^ Symptoms like lymphadenopathy, head and neck masses, and seizures, showed high positive predictive values for specific cancers, with certain indicators significantly increasing the likelihood of cancer diagnoses. However, despite increased consultation frequency, the absolute risk of cancer in TYAs who visited their GP four or more times in three months was relatively low (1.8 per 10,000), underscoring the complexity of diagnosing cancer based on non-specific symptoms. This highlights a need for education in primary care regarding the complexities and investigation thresholds for younger patients.

Ethnicity significantly influences prostate cancer diagnosis following prostate-specific antigen (PSA) testing in primary care, as demonstrated by an analysis of English primary care-linked data.^46^ The study uncovered that Black and mixed ethnicity men without prostate cancer exhibited higher PSA values than their counterparts, particularly those over 60. Within 12 months of a raised PSA, Black men had a 24.7% risk of prostate cancer incidence, compared to 19.8% for White men and 13.4% for Asian men. Late-stage diagnosis did not show significant differences between Black and White men. As PSA levels and prostate cancer risk increase with age, Black men are more likely to be diagnosed with prostate cancer. These findings underscore the importance for clinicians to consider ethnic disparities when interpreting PSA results and making decisions regarding further investigations. Additionally, research has shown that Black men have a higher likelihood of undergoing PSA testing, leading to early-stage diagnoses.^47^, ^48^, ^49^

Approximately 18–23% of cancer diagnoses occur through emergency presentations, often associated with advanced disease stages and frailty.^50^ Numerous studies across various tumour sites consistently show poorer survival rates associated with emergency presentations, highlighting potential missed diagnostic opportunities at primary, secondary care, and throughout the diagnostic pathway.^51^^,^^52^ Factors such as gender, age and ethnicity can heighten the risk of cancer diagnosis through emergency routes, with variations based on cancer type and deprivation levels.^53^ For example, higher rates of emergency presentation are reported for colorectal cancer in females, older individuals, ethnic minorities, those with multiple comorbidities, and individuals from more deprived areas.^54^

A study analysing data from over 240,000 patients through the Clinical Practice Research Datalink, found significant ethnic variations in cancer diagnosis routes.^14^ Patients fell into six groups: emergency, elective GP, 2-Week Wait (2WW), screening, hospital, or other. Most cancer cases were diagnosed through the 2WW (36.4%), followed by elective GP referral (23.2%), emergency presentations (18.2%), hospital routes (10.3%), and screening (8.6%). Notably, individuals classed as ‘other’ ethnicities had the highest likelihood of emergency department diagnosis (28.1%), comprising individuals outside White, Asian, Black, or mixed categories. Black, Asian, and mixed ethnicities generally had lower odds of emergency department diagnosis compared to Whites. Black patients were more frequently diagnosed through elective GP referrals (31.1%) compared to White (22.9%), with higher odds of 2WW diagnosis for myeloma, breast or prostate cancer but lower odds for oesophageal and oral cancers. Screening uptake was lower among Black (5.1%) and ‘Other’ (5.0%) patients compared to White (8.3%), Mixed (9.5%), or Asian (10.9%) groups, reflecting disparities in screening uptake.

Certain symptoms that meet urgent referral criteria are associated with shorter diagnostic intervals, but longer intervals are observed for specific cancers, particularly in older individuals and females.^55^ Ethnic disparities in diagnostic intervals exist, although evidence is limited and subject to methodological challenges.^1^ Additionally, a study investigating primary care visits among TYAs diagnosed with cancer found that those who were diagnosed had significantly more consultants in the 12 months leading to diagnoses compared to controls, with 87% visiting their GP in the three months prior to diagnosis, compared to 39% of controls.^45^^,^^56^

Evidence has shown that people with certain pre-existing chronic diseases have longer diagnostic intervals and more advanced stages of cancer at diagnosis.^57^ Neurological, pulmonary, cardiac and psychiatric conditions can impact participation in cancer screening or, help-seeking for new or changing symptoms, while gastrointestinal musculoskeletal conditions, or hypertension, may lead to timely help-seeking and diagnosis. A systematic review confirmed that on average, 33.4% of patients with a cancer diagnosis had comorbidities, with lung and colorectal cancer patients having the highest prevalence of comorbid conditions, such as hypertension, pulmonary diseases and diabetes.^58^

A study in England showed that mixed, Black and Asian patients with cancer were more likely to have visited their GP on three or more occasion before being referred for further investigations, irrespective of age, sex or SES.^56^ The study also found that the likelihood of requiring three of more consultations varied by cancer type and patient demographics. After multivariable analysis, patients with cancers like multiple myeloma, pancreatic, stomach and lung cancer were more likely to have had three or more consultations before referral, whereas breast cancer, melanoma, testicular and endometrial cancer were more likely to have been referred after just one or two appointments. The likelihood of multiple consultations was greater in young patients, ethnic minorities and women, highlighting the need for educational initiatives in primary care regarding differences in culture, symptom reporting and cancer incidence across populations.

Bias in doctor–patient interactions can contribute to delays in cancer diagnosis, often stemming from subconscious stereotypes or assumptions related to a patient's ethnicity, gender, or socioeconomic status. Such biases may cause doctors to minimise or dismiss symptoms presented by marginalised groups, delaying necessary tests or referrals. Additionally, linguistic and cultural communication barriers can exacerbate misunderstandings. These biases, whether implicit or explicit, lead to disparities in the timeliness and quality of cancer diagnosis for underrepresented populations. Despite the importance of this issue, no studies were found addressing these biases in the reviewed literature, highlighting an area for future research.

Nearly half of all cancer diagnoses are made through the 2WW (Two-Week Wait) pathway; however, 92% of those referred via this route ultimately do not have cancer.^59^ Non-attendance at secondary care appointments, especially by those scheduled through the 2WW pathway, remains a significant concern, with approximately 5–7% of patients either cancelling or failing to attend. A review highlighted that reasons for non-attendance included logistical challenges such as short-notice appointments, outdated contact information, and transport difficulties. Additionally, patients often cited fear of tests, anxiety about a potential cancer diagnosis, and a tendency to downplay symptoms. Personal and social factors, including multiple health conditions, mental health issues, and financial struggles, particularly among those from deprived backgrounds, also contributed to missed appointments. Effective communication between GPs and patients was crucial, as misunderstandings and a lack of clarity about the urgency of these appointments significantly influenced patient decisions.

Another study found that geographical location posed a significant barrier to accessing cancer care, as treatment facilities are often concentrated in urban areas and affluent areas.^60^ This centralisation disproportionately affects people from deprived areas, who may face additional out-of-pocket expenses for travel and transport, leading to missed appointments, treatment delays, or even an inability to receive treatment. Radiotherapy treatments, which requires multiple visits over days or weeks, are particularly affected, with transport difficulties contributing to poor treatment adherence and extended treatment durations among disadvantaged populations.

A review of cancer treatment for incarcerated individuals revealed numerous complex barriers to diagnosis and care.^61^ Communication challenges, restricted patient autonomy, and logistical difficulties were common, particularly with the prison medical requesting system, which requires inmates to use precise language to expedite appointments. Post-diagnosis, communication between specialists, patients and prison staff were minimal. Issues related to transport, safety protocols and medical confidentiality further exacerbate these challenges for patients managing complex treatment regimens while incarcerated.

## Screening

Cancer screening is discussed separately from the rest of the Anderson model because it fundamentally differs in how it influences delays in the diagnostic process. Screening reduces both appraisal and illness delays by detecting cancer before symptoms even emerge, effectively bypassing the need for patients to recognise and act on potential warning signs.^20^

### Breast cancer

In the UK, all cancer screening programmes aim to achieve an 80% coverage rate. Despite this target, breast cancer screening rates have remained steady since 2012. Various barriers impact screening participation, particularly among people from ethnic minorities and socioeconomically deprived areas. Exploring breast cancer screening perceptions among British-Pakistani women, for example, revealed cultural taboos, the importance of privacy and modesty, and beliefs that influenced their views on screening.^62^ Trust in GPs emerged as vital, emphasising the importance of community collaboration with healthcare providers to endorse screening. Similarly, South Asian breast cancer patients reported the need to discuss health issues outside their homes due to perceived restrictions, highlighting the influence of cultural norms on healthcare-seeking behaviour.^63^

Physical disabilities can also hinder screening attendance due to transport challenges and accessibility issues.

This can further be compounded by social deprivation, and has been observed in lower breast cancer screening uptake in several trials.^64^ A review in Northern Ireland found a 7% lower attendance rate among women with physical disabilities in breast cancer screening, highlighting the need for improved accessibility and tailored support.

### Cervical cancer

Cervical screening attendance has declined, particularly among minority ethnic groups and in more deprived areas.^51^^,^^62^^,^^65^ This trend is often attributed to cultural beliefs and a lack of awareness regarding cancer signs and screening programmes, contributing to negative perceptions. Common barriers to screening participation include poor literacy and numerical skills, which disproportionately affect ethnic minorities and lead to reduced uptake and less positive attitudes toward screening.^51^ A study, employing multi-lingual interviews, found that ethnic minorities preferred strong screening recommendation (53–86%), contrasting with 31% of White British participants.^66^ Socioeconomic factors and culture played a role, but ethnicity remained significant after multivariate logistic regression analysis. Individuals born outside the UK and those migrating as adults sought stronger recommendations (78% vs. 45%, p < 0.001). Non-English speakers favoured strong recommendations (85% vs. 49%, p < 0.001). It seems logical therefore to consider different wording of screening invitations based on the individual in mind. This relies on accurate documentation of ethnicity of participants’ ethnicity, and this being shared from primary care to screening services.

Cervical screening attendance is declining in young UK women, despite a rising incidence of invasive cervical cancer.^51^ In addressing barriers to cervical screening, a study aimed at young women identified factors such as embarrassment and inconvenience.^67^ Preferences for interventions varied based on test location, nurse availability, and cost, with unsolicited screening, particularly the Human Papillomavirus (HPV) self-sampling kit, emerging as the most favoured option. However, the low 6% response rate limits generalisability, and it overlooks the potential impact of the HPV vaccine on attitudes and behaviours.

The current NHS cervical screening programme excludes gender minorities assigned female at birth (AFAB), encompassing trans-men and non-binary individuals.^16^ Trans-men are those assigned female at birth but identifying and living as men, while non-binary refers to those whose identity doesn't align strictly with ‘man’ or ‘woman’. AFAB gender minorities are not automatically invited for cervical screening if registered as male at their GP, with Scotland making exceptions only for those who transitioned post-June 2015. A systematic review of 27 papers revealed significant disparities in cervical cancer screening uptake between AFAB gender minorities and cis-women (someone who was assigned female at birth and identifies as a woman). AFAB gender minorities exhibited lower odds of both lifetime and up-to-date screening. Influencing factors include a lack of trans-specific resources, evidence-based guidelines, gender dysphoria, and the effects of androgen therapy. The study underscores the inequality in cervical cancer screening invitations, stressing the need for understanding and education to enhance uptake among AFAB gender minorities.

### Bowel cancer

Individuals with learning disabilities face greater health problems than their non-disabled peers, with an average life expectancy of 15 years less than the general population.^6^^,^^68^ People living with learning disabilities often face challenges in accessing cancer screening programmes due to various barriers, including literacy issues and difficulties with home testing.^68^^,^^69^ While the incidence of cancers is generally similar between individuals with and without learning disabilities, certain syndromes are associated with specific cancers.^69^ Men with Down's syndrome may have a higher risk of testicular cancer, and there is some indication that women with learning disabilities exhibit a lower incidence of breast cancer. However, these findings may be influenced by low screening uptake and historically lower life expectancy in this population. Individuals with learning disabilities have an elevated risk of bowel cancer possibly due to factors such as obesity, poor diet, and lack of exercise.^68^

Bowel cancer screening uptake is only 26% in individuals with learning disabilities, compared to 39% in the general population. Similarly, cervical and breast cancer screening uptake is significantly lower in this group, standing at 27% and 38% respectively, compared to 70% and 63% in the overall population.^69^ Learning disability nurses play a crucial role in addressing these barriers through education and support and collaboration with relevant health professionals to enhance screening participation. Evidence suggests no increased cancer risk in individuals with severe mental illness. However, our knowledge is hindered by small scale studies or insufficient consideration of confounding factors.^70^

### Lung cancer

Targeted lung cancer screening using low-dose Computed Tomography (CT) scans has shown promise in reducing mortality, with equitable uptake across diverse ethnic and socioeconomic groups being a key aim. The SUMMIT study in London found that of 95,000 eligible participants, 31% responded, with an additional 14% responding after a further reminder after 4 months.^71^ Overall, lower uptake was noted in younger participants, the more deprived, and current smokers. Examining ethnicity, ‘other White’ participants had the lowest uptake, while Chinese, Indian, and other Asians showed high participation compared to White British. Former smokers had higher uptake than current smokers.

A review undertaken to identify interventions to increase targeted lung cancer screening, particularly in those from more deprived areas, women and current smokers found 36 interventions.^72^ These focused on increased cancer awareness, improved sociocultural acceptability, improved availability of screening, reduced financial barriers and encouraging patient decision-making. Key strategies included shared decision-making, education, community-based campaigns and smoking cessation support. Of note, community-based interventions that did not rely on previous engagement with the healthcare system were particularly successful.

## Outstanding questions

A key area for further consideration is how cancer prevention and screening campaigns can more effectively target the specific challenges faced by deprived populations and ethnic minorities. These groups often encounter barriers such as lower health literacy, stigma, fatalism, and financial burdens, which limit their access to early diagnosis and treatment. Campaigns must consider these multifaceted issues and focus on reducing stigma while improving awareness and support networks.

Another pressing question concerns the best strategies for reaching marginalised groups such as rough sleepers, migrants, individuals with substance abuse issues, and those involved in the justice system. These populations are often overlooked in health interventions, and more research is required to understand their unique challenges and develop tailored approaches that meet their specific needs. The disparities in cancer care access and outcomes within these groups must be addressed through comprehensive, targeted initiatives.

Additionally, there is a critical need for healthcare systems to improve data collection and ensure that cancer care disparities are accurately identified across all minority and marginalised groups. Current datasets often lack the necessary granularity to reflect the full diversity of the population, hindering the development of effective interventions. Updated and precise data will enable a more thorough assessment of current practices and guide strategies to enhance care standards.

Finally, the role of local determinants in creating disparities for LGBTQ + individuals and people with disabilities warrants further investigation. Local barriers to healthcare access, such as discrimination or lack of culturally competent services, can exacerbate inequalities. Understanding these local dynamics is essential for tailoring interventions to reduce healthcare disparities in specific communities.

## Conclusion

While progress has been made in cancer care, significant disparities remain, especially among deprived and marginalised populations. Addressing these inequities requires culturally sensitive care, targeted interventions, and improved data collection to guide policy and practice. Ongoing research is essential to close these gaps and ensure equitable access to cancer services for all individuals, regardless of their background or circumstances.

## Limitations

This review faced challenges due to the varying methodologies used across studies, especially in defining and categorising ethnicity, socioeconomic status, and health literacy, making it difficult to draw consistent conclusions and compare results. Many studies relied on retrospective data, which is prone to recall bias and may not accurately represent real-time health behaviours. Additionally, the review is constrained by selection, interpretation and publication biases, and is limited to studies published before June 2024, reducing its replicability.

## Recommendations

To enhance early cancer diagnosis with the biggest effect, efforts should focus on improving public awareness and education through culturally sensitive campaigns tailored to diverse ethnic groups. Examples of good practice can be seen from the ‘Be Clear on Cancer’ campaign, and ‘Get on a Roll’ from Bowel Cancer UK. Implementing school-based programmes can increase cancer prevention and symptom awareness among adolescents, but it important to ensure long-term sustainability and impact. National campaigns should target cancer-related stigma and fatalistic beliefs, particularly in minority and lower socioeconomic groups. Support groups and counselling services for patients and families can address fears and emotional barriers linked with a cancer diagnosis and treatment.

The next largest impact can stem from strengthened community engagement by closer working between Integrated Care Systems, Local Authorities and local leaders to promote cancer awareness and screening in minority populations. Mobile screening units and community-based health clinics can increase accessibility in underserved areas. Culturally appropriate screening invitations with different wording or phrasing for different cultures, and its availability in more languages. Invitations can also include educational materials and should emphasise the importance of screening and empower patients, for example, to request female healthcare workers. The potential offer of flexible screening opportunities such as home testing kits will improve screening uptake with privacy or convenience barriers removed.

Healthcare service accessibility, including translation services and culturally competent healthcare navigators should be available to overcome understanding and access in those with language barriers. Expanding telemedicine can enhance primary care access, particularly in remote and deprived areas. Financial assistance and transport services can reduce barriers to screening and treatment for low-income individuals and those in remote areas and improve accessibility for those with physical difficulties.

Individuals with disabilities need better support, including specialised nurses and accessible healthcare facilities. The healthcare system needs to improve training and awareness about learning, physical and mental health difficulties and its impact on patients in the NHS, and to mandate cultural competency training. There is currently little training for healthcare professionals on cultural sensitivity, implicit biases, and effective communication strategies to improve interactions with diverse patient populations. The current 30-min e-learning module, free to access for all healthcare professionals on the NHS England portal, is not mandatory for most professionals and has limited impact on practice. Cultural competency training run by services outside the NHS can be tailored to the specific needs of staff within the NHS.

National Cancer Control Plans and regional Cancer Alliances must align on health literacy initiatives to ensure a cohesive approach to reduce cancer incidence and mortality. Improving data collection, amalgamation and access to data for those in charge of strategy and finances, identifies future research channels and informs targeted interventions.

## Contributors

**EmilyC.S.Scott:** Formal Analysis, Investigation, Methodology, Writing Original Draft, Approve final manuscript.

**PeterJ.Hoskin:** Conceptualisation, Supervision, Review & Editing, Approve final manuscript.

## Date sharing statement

No new data were created or analysed in this review. Data sharing if therefore not applicable.

## Declaration of interests

Emily C.S. Scott: None.

Peter J. Hoskin: None.
